# Dynamic regulation of RAS and RAS signaling

**DOI:** 10.1042/BCJ20220234

**Published:** 2023-01-06

**Authors:** Walter Kolch, Dénes Berta, Edina Rosta

**Affiliations:** 1 Systems Biology Ireland, School of Medicine, University College Dublin, Belfield, Dublin 4, Ireland; 2 Conway Institute of Biomolecular & Biomedical Research, University College Dublin, Belfield, Dublin 4, Ireland; 3 Department of Physics and Astronomy, University College London, Gower Street, London WC1E 6BT, U.K.

**Keywords:** biological networks, cancer, dynamics, RAS proteins, signaling

## Abstract

RAS proteins regulate most aspects of cellular physiology. They are mutated in 30% of human cancers and 4% of developmental disorders termed Rasopathies. They cycle between active GTP-bound and inactive GDP-bound states. When active, they can interact with a wide range of effectors that control fundamental biochemical and biological processes. Emerging evidence suggests that RAS proteins are not simple on/off switches but sophisticated information processing devices that compute cell fate decisions by integrating external and internal cues. A critical component of this compute function is the dynamic regulation of RAS activation and downstream signaling that allows RAS to produce a rich and nuanced spectrum of biological outputs. We discuss recent findings how the dynamics of RAS and its downstream signaling is regulated. Starting from the structural and biochemical properties of wild-type and mutant RAS proteins and their activation cycle, we examine higher molecular assemblies, effector interactions and downstream signaling outputs, all under the aspect of dynamic regulation. We also consider how computational and mathematical modeling approaches contribute to analyze and understand the pleiotropic functions of RAS in health and disease.

## Introduction

RAS is a family of small G-proteins that became famous as one of the first oncogenes to be discovered and infamous as the bully amongst the oncogenes causing aggressive cancers that defy treatment. Almost 20% of all human cancers and 4% of developmental disorders feature mutations in one of the three RAS genes (KRAS, NRAS or HRAS) [1]. This made RAS a prime target for research and drug development [2]. Only now we are closing in on understanding RAS biology and how we can exploit it for drug development and better treatment options.

**Figure 1.**
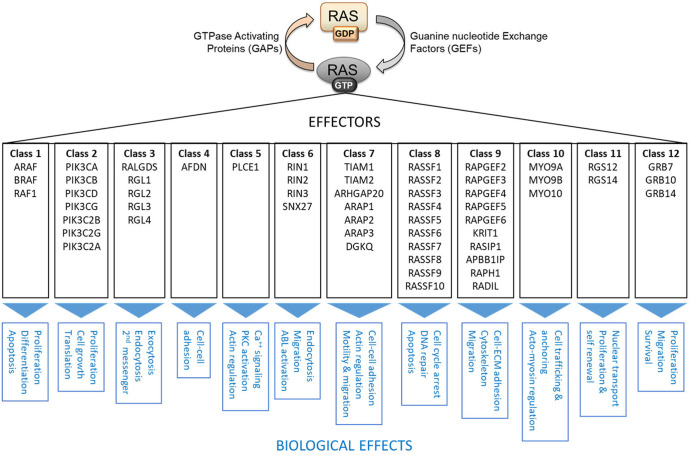
RAS activation cycle and RAS effectors. Fifty-six bona fide RAS effectors can be grouped into 12 classes according to sequence homology and regulation of downstream biochemical and biological processes. Adapted from [5].

It all looked very simply. Ras sits at the apex of a cascade of three kinases RAF–MEK–ERK, which drive oncogenic transformation [3]. Thus, blocking this pathway should obliterate RAS transformation. Unfortunately, it did not [2]. A plausible reason is that in addition to the RAF–MEK–ERK pathway RAS has 56 bona fide effectors, which can modulate RAS signaling [4, 5] (Figure 1). Another, related reason could be different dynamic regulation. RAS regulates its downstream effectors by directly binding them through a single ‘effector binding domain’, which causes an elaborate control of effector binding by competition, binding affinities, abundances and subcellular localization [5]. This highly dynamic scenario of RAS effector interactions gives rise to intricate downstream signaling effects that impact all fundamental processes of living cells.

**Figure 2.**
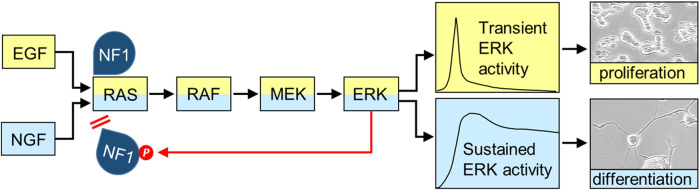
RAS activation kinetics determine cell fate decisions in PC12 cells. PC12 cells proliferate in response to EGF, which causes transient RAS and ERK pathway activation, whereas NGF causes sustained RAS and ERK activation and neuronal differentiation (visible as the extension of long neurites). Feedback phosphorylation of the NF1 GAP by ERK occurs selectively in response to NGF stimulation [9]. It interferes with NF1 binding to RAS thereby allowing RAS activation to persist.

Life is a continuing transition between dynamic states. Thus, it is not surprising that signal transduction networks (STNs) exploit dynamics for signal processing [6]. An early and now classic example are the differential effects of EGF and NGF on PC12 cells [7,8]. EGF causes a transient activation of the RAS–ERK pathway stimulating proliferation, whereas NGF induces sustained RAS and ERK activities and differentiation (Figure 2). Later analysis of this phenomenon revealed that these differences in activation kinetics are regulated and interpreted through various processes throughout the RAS STN [9]. These processes involve changes in protein–protein interactions (PPIs), subcellular localization and enzymatic activities that co-operate to produce the specific biological outcomes. These biological decisions usually require changes in gene expression, and the transcriptional machinery is often considered as a decoding device that converts peripheral signaling dynamics into differential gene expression patterns. For instance, the simultaneous monitoring of RAS activity and the expression of five immediate early genes (IEGs) showed that transcription functions as a bandpass filter that optimizes gene expression for ERK activity pulses that are frequent enough to trigger transcription, but not too sustained to invoke negative feedback mechanisms [10].

This review focuses on the dynamics of RAS signaling and its impact on downstream biological and pathological effects. It will discuss RAS dynamics starting from the biochemical protein properties, then considering its PPI partners and biophysical environment to further downstream effects. Finally, we will highlight open questions. The review does not consider current efforts to develop RAS inhibitors as this topic has been extensively reviewed in recent years [11–16].

## Dynamic regulation of RAS activity operating at the GDP–GTP exchange cycle

**Figure 3.**
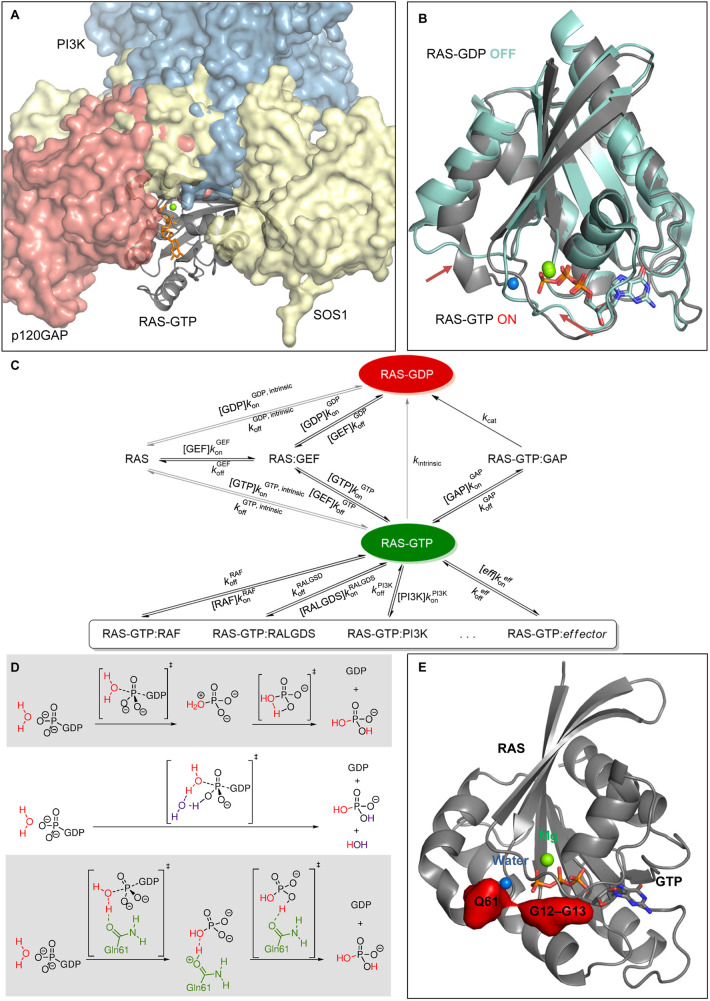
RAS protein activation cycle. (**A**) Complex of p120GAP (red surface, PDB 1WQ1), SOS1 (yellow surface, PDB 6V6M) and the effector PI3K (blue surface, PDB 1HE8) showing a shared binding interface with GTP loaded RAS (gray cartoon, PDB 1QRA). (**B**) Comparison of GTP-bound RAS (gray cartoon, PDB 1QRA) and GDP-bound RAS (cyan cartoon, PDB 6P0Z), conformational changes of Switches I and II are highlighted by arrows. (**C**) Kinetic network of the RAS signaling cycle and downstream signaling. Adapted from [19]. (**D**) Alternative proton transfer mechanisms in the GTP hydrolysis. Adapted from [32]. (**E**) Oncogenic mutation hot spots (red surfaces) highlighted in the GTP-bound RAS structure (gray cartoon, PDB 1QRA).

RAS proteins are traditionally considered molecular switches that cycle between an inactive GDP-bound and an active GTP-bound form that can interact with downstream effectors [2]. GTP binding changes the conformation of RAS enabling it to bind its effectors via a single shared binding site, the effector domain (Figure 3A). The critical conformational event is the rearrangement of the so-called Switch I and II regions that together form the effector domain. The signaling dynamics of RAS proteins is thus regulated by three types of rates: (i) GTP hydrolysis, (ii) nucleotide binding/unbinding and (iii) effector binding/unbinding. Hydrolysis of GTP to GDP is facilitated by GTPase activating proteins (GAPs) that increase the intrinsic catalytic rate by 2–5 orders of magnitude [17–19] and return RAS into the inactive conformation (Figure 3B). Release of GDP mediated by guanine nucleotide exchange factors (GEFs) causes the loading of RAS with GTP, as the intracellular concentration of GTP is 10- to 20-fold higher than that of GDP. Rate acceleration by GEFs and GAPs, therefore, provide the mechanism for switching signaling on or off, respectively, and open up the competition for downstream effector binding (Figure 3C).

Structural data of RAS and its interaction partners are typically available, albeit limited and unbalanced. Reflecting the weaker affinity of GAPs to RAF compared with GEFs, there are ∼10 times more structures of RAS bound to GEFs, than RAS–GAP complexes. Only SOS1 and RASGRP1 crystal structures represent GEFs, and two major canonical GAPs, p120GAP and NF1 have available complexes with RAS. The most studied effector is RAF, but several others RAS effector complex are also resolved: Afadin, RALGDS, Byr2, NORE1A, Grb14, PLCε, PDEδ, Shank3 and SIN1. Typically, however, only the effector's RAS-binding domain (RBD) is resolved, with a notable exception of PI3K [20]. Additionally, more transient binding interfaces with regulatory roles were also proposed for some effectors. SIN1 has an atypical SIN1-RBD [21], and an additional interface was suggested to modulate effector binding. Titration experiments show that effector-RAS binding can both be enhanced and diminished by oncogenic mutations [22]. More recently, the attention turned to synthetic constructs [23–25] and non-natural binding partners [26–28], some of which demonstrate binding interfaces that go beyond the biological Switch I and II regions. However, a complete functionally competent structure of RAS is still elusive. This is largely due to the lack of information on membrane-bound assemblies, which is the native cellular environment of RAS and involves its dimerization and/or oligomerization.

Oncogenic RAS mutations generally impair GTP hydrolysis. Both the intrinsic and GAP-mediated phosphate cleavage reaction by RAS proceeds via an associative phosphate cleavage with a loose transition state [29–31]. However, the crucial, likely rate-determining coupled proton transfer that deprotonates the nucleophilic water and forms the inorganic phosphate (Figure 3D) remains controversial [32–34]. The majority (∼97%) of the oncogenic mutations are concentrated at only three hot spots [35]: G12 and G13 in the P-loop and Q61 in Switch II, all in close proximity to the reaction site (Figure 3E), with Q61 most likely involved in the proton transfer [32]. Oncogenic mutations can impair the GTPase activity of the RAS–GAP complex, although the precise mechanisms are not yet established. Interestingly, different GAPs can have varying hydrolysis rates on specific RAS mutants. For instance, NF1 is more active on RAS G13D than p120GAP [36], and RGS3, a non-canonical GAP, retains significant activity on RAS G12C [37].

## RAS conformational dynamics and effect of mutations

The cycling between inactive and active conformation that is disturbed by oncogenic mutations itself is a rich source for dynamic variation. Extensive molecular dynamics (MD) simulations were used to compare the cycling of three RAS isoforms [38]. The differences observed between RAS isoforms are due to different flexibilities in the Switch I and II regions, which can further change depending on whether GDP or GTP is bound. These simulations also identified a pocket that transiently opens during the cycling and which is similar between HRAS and NRAS but smaller in KRAS [38]. Thus, isoform-specific inhibitors could be designed. MD simulations also identified Switch I conformational states optimal for RAF versus GAP binding [39]. Others focused on the GEF-induced disruption of Mg^2+^ binding, which leads to enhanced nucleotide exchange [40]. Although MD simulations are very useful in analyzing reaction scenarios and guiding experimental work, one has to keep in mind that they are models that require experimental validation.

Oncogenic mutations, such as G12C, G12V, G13D and Q61H, change the flexibility of Switch I and II as well as their conformational motions [41–43]. This can affect effector binding in addition to the GDP–GTP exchange cycle by inducing slightly different conformations of the nucleotide-binding site and the effector-binding site. Mutations can also affect the rates of binding and unbinding events, e.g. G13D and Q61L mutations enhance the intrinsic nucleotide exchange rate, while G12D slows it down [44,45]. GDP-bound by KRAS G12C or G12D mutants is more accessible than when bound to wild-type KRAS, which facilitates GDP to GTP exchange and activation. Q61 mutations further subvert GAP function to stabilize the active KRAS state rather than hydrolyzing the activating GTP [42]. They influence the distribution of water molecules in the active site [46] disrupting the catalytic water deprotonation.

Although effector binding is structurally less affected by mutations, its dynamics is impacted. Recent crystallographic structures of G12V, G13D and Q61R present very similar RAS–RAF binding interface between wild-type and mutant RAS [47]. Q61L has a slightly altered RAF binding interface and different loop flexibility [48]. Sophisticated NMR techniques revealed synchronized sub-millisecond conformational motions in KRAS that link the effector and allosteric domains [49]. Mutations that disrupt this coupling substantially reduce the binding of the RAF1-RBD [49]. Effector-specific affinity can also change upon mutations. For example, the Q61K mutation enhances the binding of RAF while diminishing the binding of PI3K [50]. Conceivably, these dynamic conformational changes could change downstream signaling, and also could be exploited to design drugs that differentially interfere with effector binding in wild-type and mutant RAS proteins [28,51].

**Figure 4.**
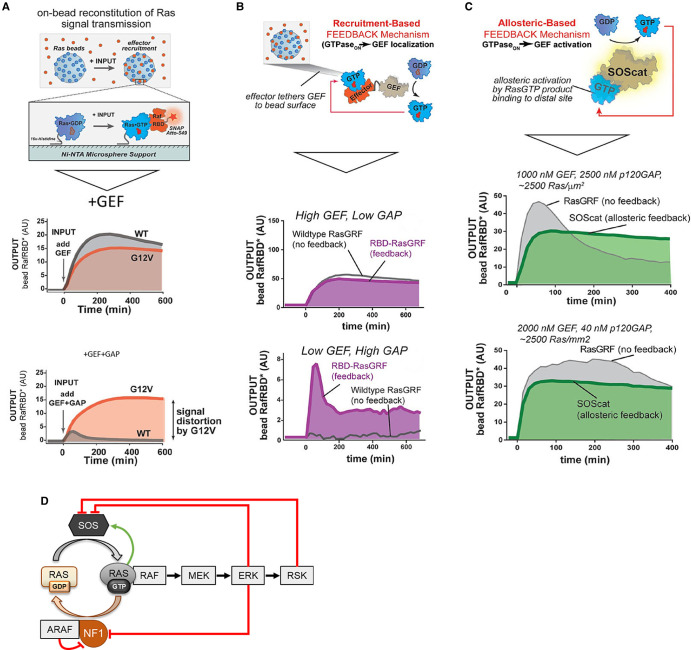
Dynamic RAS regulation by GEFs and GAPs. (**A**) RAS immobilized on microbeads is used to study the effects of GEFs and GAPs on RAS activation as measured by the recruitment of a labeled RAF1-RBD. (**B**) A positive feedback that recruits more GEF (RBD-RasGRF) to beads as more RAS is activated amplifies weak input signals under high GAP conditions. (**C**) The allosteric feedback whereby RAS:GTP binds to and activates SOS reduces overshoot in the output dynamics. Panels (**A**–**C**) are adapted from [52]. (**D**) Feedback regulation of SOS and NF1. Green arrow, activation; red blunt-end lines, inhibition.

Thus, the dynamic nature of the GDP/GTP exchange cycle already provides a rich source for dynamic regulation. Coyle and Lim [52] used a reductionist *in vitro* system consisting of recombinant purified proteins to quantify the effects of GEFs and GAPs on HRAS activity. They found that HRAS activation as measured by binding of the RAF1 RBD to HRAS immobilized on beads was highly dependent on the abundance of added SOS1-GEF and GAPs (Figure 4A–C). Increasing GAPs decreased the response amplitude but sharpened the response peak with the NF1 GAP resulting in tighter peaks than p120GAP. These properties allowed HRAS activation to overshoot in the presence of NF1 before equilibrating at a lower steady state indicating that the biochemical properties of GAPs shape the dynamic response. There was also a difference between RAF1 and BRAF RBDs with the latter showing more and more sustained binding. Introducing a recruitment-based positive feedback through tethering the GEF to the RBD so that it could act directly on the HRAS bound to beads, amplified weak GEF signals under high GAP conditions. Allowing a more physiological positive feedback, i.e. the allosteric activation of SOS1 by GTP [53], reduced the overshoot in HRAS activation caused by a the RasGRF GEF which has no allosteric positive feedback regulation. Increasing the RBD concentration also led to a higher activation amplitude with transient peaks that slowly decayed to a lower steady state. Interestingly, a HRAS G12V mutant still was responsive to GEF-mediated activation but refractory to GAP-mediated deactivation showing a steady increase in activity without overshoot. This analysis showed that rich dynamic behavior can already be encoded by changing the cycling of HRAS between GTP and GDP-bound states. Thus, depending on the abundance of different GEFs, GAPs and effector proteins a single input stimulus can elicit a variety of RAS activation patterns that presumably also propagate into the regulation of downstream signaling.

Disturbing these balances has pathophysiological consequences. Noonan syndrome belongs to a family of developmental disorders, called Rasopathies, which are hallmarked by hyperactive RAS signaling [1, 2]. This is due to genetic variants in RAS pathway genes including a series of RAS variants. Some of these RAS variants, namely I24N, T50I, V152G and D153V, use the allosteric activation of SOS1 to enhance their activity [54]. They bind to the SOS1 allosteric site to activate SOS1 and promote their own activation. SOS1 is also subjected to negative feedback phosphorylation by ERK and its downstream kinase RSK which impairs membrane recruitment of SOS1 and subsequent RAS activation (Figure 4D) [55–59]. A computational modeling study suggested that effective inhibition requires multiple SOS1 phosphorylation [60]. This conceivably could act as a timing device, where only sustained ERK activity causes SOS1 inhibition and down-regulation of RAS activation.

A mathematical model that integrated both positive and negative feedback regulation of SOS showed that the switch-like activation conferred by the positive feedback is converted into oscillations by the negative feedback [61]. Interestingly, such intercalated feedback loops can give rise to local excitation/global inhibition (LEGI) dynamics, where a fast positive feedback targeting slow diffusing membrane proteins is coupled to a slower negative feedback exerted by faster diffusing cytosolic components. LEGI can perceive non-uniform input signals, such as localized growth factor stimulation resulting from paracrine cell–cell interactions, and generate a traveling wave of activated RAS that spreads over the plasma membrane (PM). Such mechanisms can interpret spatial cues and could be important for sensing gradients of growth factors and cytokines.

## Spatiotemporal and posttranslational control of RAS signaling

RAS proteins are traditionally viewed as PM proteins, but their lipid modifications enable them to diffuse to endomembranes. To keep RAS proteins in place, the cell has active transport and removal processes that put RAS proteins at the appropriate destinations and remove them before they can diffuse [62]. Not surprisingly, this compartmentalization of RAS proteins is also exploited for the dynamic regulation of RAS signaling. A critical observation was that RAS can signal from different domains in the PM, from the Golgi apparatus (GA), and from the endoplasmic reticulum (ER), engaging different signaling pathways from different subcellular locations [63–66]. Thus, one can envision co-ordinated waves of differential signaling emanating from distinct sites of RAS activation. For instance, PLCγ signaling causes a fast and transient activation of RAS at the PM and a delayed but sustained RAS activation at the GA. These dynamics are co-ordinated by PLCγ translocating the GEF RASGRP1 to the GA, while activating the GAP CAPRI at the PM [66]. This integration between signaling kinetics and differential pathway activation through subcellular compartmentalized and timed activation provides an attractive mechanism for controlling biological processes that require sequential activation events.

**Figure 5.**
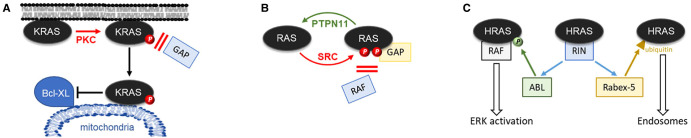
RAS regulation by posttranslational modifications. (**A**) PKC phosphorylation of KRAS at S181 sustains activation by preventing GAP binding and induces mitochondrial translocation of KRAS, where it promotes apoptosis by binding to and inhibiting the function of the anti-apoptotic Bcl-XL protein. (**B**) SRC phosphorylation of tyrosines 32 and 64 inhibits RAS by interfering with RAF binding and promoting association with GAPs. (**C**) The RIN1 effector can stimulate the ABL kinase to phosphorylate tyrosine 137 in HRAS promoting activation and enhanced RAF binding with subsequent ERK activation. On the other hand, RIN1 can stimulate the ubiquitin ligase Rabex-5, which mono-/di-ubiquinates HRAS leading to endosomal sequestration and HRAS inactivation.

Posttranslational modifications introduce a further twist on this type of control (Figure 5). Protein kinase C (PKC) can phosphorylate S181 in the KRAS polybasic region that mediates PM association [67]. The phosphorylation translocates KRAS from the PM to the ER, GA, and the mitochondria where it binds to Bcl-XL and subverts the survival function of this protein. In addition, PKC phosphorylation interferes with KRAS deactivation by calmodulin [68] and by GAPs resulting in enhanced transforming capabilities [69]. Thus, PKC can modulate both KRAS activation and signaling specificity.

The phosphorylation of activated RAS proteins by SRC family kinases on tyrosines 32 and 64 increases GAP binding and RAS deactivation [70,71]. This inhibitory phosphorylation is removed by the SHP2 phosphatase [72]. SHP2 also plays a key role in the development of resistance to KRAS G12C-specific inhibitors by enabling RTK-mediated feedback activation of wild-type RAS. Combining KRAS G12C with SHP2 inhibitors breaks this feedback resulting in sustained RAS inhibition [73]. In contrast, the phosphorylation of HRAS tyrosine 137 by the ABL kinase enhances HRAS activity and downstream signaling [74]. This regulation seems part of an intricate feedback loop. HRAS can activate ABL via RIN1, a direct RAS effector [74]. However, RIN1 also stimulates HRAS ubiquitination and sequestration in endosomes [75]. Thus, RIN1 could co-ordinate HRAS activation with subsequent inhibition. The important function of ubiquitination for RAS regulation has been recently reviewed [5,76] and, therefore, is not further discussed here.

In addition, SOS1 recruitment to the cell membrane is dynamically regulated. In response to RTK activation SOS1 can be recruited by different mechanisms [77]. They all involve a GRB2–SOS1 core complex that can either bind directly to activated receptor tyrosine kinases (RTKs) or indirectly access the cell membrane via binding to the GAB1/2 scaffold or the SHC1 adaptor. We currently do not have a clear picture about these different recruitment mechanisms. A plausible possibility is that they enable differential dynamic regulation. The RAS activation route via GAB1 is regulated by a positive GAB1–PI3K feedback and negative ERK–GAB1 feedback [77] suggesting that the GAB1 route integrates competing dynamic signals. On the other hand, SHC1-mediated SOS1 recruitment may function as a temporal ordering mechanism. EGF stimulation of Rat-2 fibroblasts triggers two waves of SHC1 phosphorylation that recruit different interacting proteins [78]. The early wave includes recruitment of GRB2–SOS1 and subsequent activation of RAS and its downstream pathways, such as RAF–MEK–ERK and PI3K–AKT. The second phase is initiated by AKT-mediated feedback phosphorylation of SHC1, which enables SHC1 to recruit the PTPN12 tyrosine phosphatase. PTPN12 displaces SHC1 from the EGFR and also dephosphorylates the SHC1 binding sites thus ending RAS activation.

Similar results were obtained in single-molecule imaging studies that tracked the recruitment of the SHC1–GRB2–SOS1 signaling complex in heregulin-stimulated MCF7 breast cancer cells [79]. The authors observed a quick recruitment and activation of RAS that was followed by a second wave of sequestration of GRB2–SOS1 in the cytosol and termination of RAS activation. An additional twist is that upon activation of the T-cell receptor GRB2–SOS1 can form liquid droplets that physically cluster signaling molecules and enhance signaling output by concentrating kinases while excluding phosphatases [80]. An *in vitro* system using reconstituted proteins showed that the *Caenorhabditis elegans* protein LAF1 can form liquid granules that incorporate KRAS as well as RAF1 and BRAF [81]. The granules can fuse with artificial lipid membranes leading to the formation of KRAS nanoclusters. It is currently unclear whether liquid phase separation plays a role in regulating RAS activation in other mammalian cell types. However, the spatiotemporal control afforded by liquid clusters seems an attractive way to regulate RAS activation kinetics in situations where a fast and high pulse of RAS activity is required such as in T-cell receptor signaling.

As may be surmised from the importance of balancing GEF and GAP activities in the *in vitro* system [52], GAPs are also subjected to feedback regulation. This mechanism is less well explored. A study analyzing RAS activation in EGF-stimulated primary hepatocytes showed that the down-regulation of SOS1 activity cannot explain the RAS activation kinetics and suggested that a transient GAP activation is needed possibly mediated by membrane recruitment of RasGAP [82]. Similarly, a study monitoring the RAS activation cycle in permeabilized cells indicated that the concentration of active GTP-bound RAS decreased despite continuing high GDP/GTP exchange rates [83]. Experimental observations and mathematical modeling suggested that feedback activation of NF1, possibly mediated by RSK1/2 kinases, is responsible for this effect [83]. On the other hand, ERK-mediated phosphorylation can inhibit NF1 binding to RAS thereby extending the duration of ERK activation (Figure 4D). In addition, NF1 also can be prevented from RAS binding by competition from the RAS effector ARAF [84] or RAS mutants that strongly bind to and sequester NF1 [85]. Both mechanisms enhance RAS activity. Thus, the kinetics of RAS activation seen *in vitro* when GEF and GAP concentrations are modulated, seem to be overlayed by dynamic feedback regulations of both GEFs and GAPs in cells.

## RAS clustering

**Figure 6.**
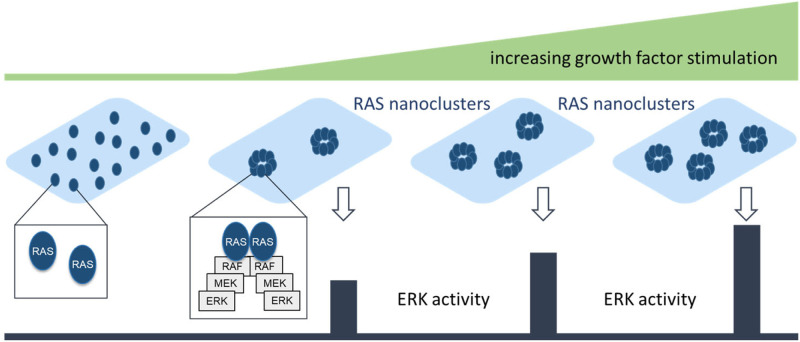
RAS nanoclusters and signaling output. Each RAS nanocluster delivers a defined ‘digital’ signaling output. Increasing concentrations of growth factor induce more nanoclusters thereby transforming an analog input of growth factor stimulation into a digital output of ERK activity.

The spatial organization of RAS proteins at the membrane is another source of generating different output kinetics (Figure 6). RAS can form nanoclusters that consist of 6–8 RAS proteins, which produce digital outputs in response to analog stimuli, such as increasing amounts of growth factors [86]. If growth factor stimulation induces a RAS nanocluster to form, it will activate the ERK pathway at a fixed rate. Increasing the growth factor concentration will increase the number of nanoclusters, but not the output of a nanocluster. This mechanism reduces the impact of noise, e.g. arising from random fluctuations of receptor activation, and translates true signals with high fidelity [86]. Extending the short lifetime of RAS nanoclusters also increases their signal output [87]. Nanocluster formation seems to be preceded by RAS dimerization, and both mechanisms have recently received much attention as they seem to be necessary for oncogenic signaling by RAS [88].

Using *in vitro* systems of purified proteins or cells expressing RAS proteins with artificial dimerization domains, RAS dimerization was found to facilitate RAF1 activation more than 20 years ago [89]. However, it currently is debated whether RAS can form dimers on its own or whether clustering is mediated by scaffolding proteins and membrane domains. At physiological concentrations, KRAS was reported to form dimers, but only KRAS proteins with an activating mutation could activate RAF1 [90]. At lower expression levels KRAS G12D becomes monomeric and unable to activate RAF1 unless artificially dimerized. At higher than physiological concentrations KRAS forms nanoclusters [90], suggesting that physiological RAS signaling is confined to a certain bandwidth of RAS expression levels. Interestingly, the expression of mutant RAS tends to increase in both tumors and tumor cell lines [91], likely a mechanism that contributes to increased clustering and signaling. Another factor is effector dimerization, which co-operates with RAS clustering to promote the interaction between RAS and effectors and may mutually enhance dimerization. RAF inhibitors that enhance RAF1 and BRAF homo- and heterodimerization also increase the dimerization of KRAS and NRAS (but not HRAS) and the formation of cognate nanoclusters [92]. The RAF dimers can outcompete non-dimerizing effectors, such as PI3K, resulting in enhanced ERK and decreased AKT activation [92]. Thus, dimerization and clustering seem an interdependent property of RAS and its effectors that does not only affect the amplitude and duration of RAS signaling but also the balance between effector pathways.

RAS nanoclustering is also regulated by membrane lipids [93]. Depolarization of the cell membrane selectively reorganizes phosphatidylserine and phosphatidylinositol 4,5-bisphosphate inducing KRAS nanoclustering and ERK pathway activation. This is due to KRAS directly interacting with phosphatidylserine. Membrane repolarization disassembles KRAS nanoclusters and terminates ERK signaling. Thus, excitable membrane processes can directly impinge on KRAS activation and downstream mitogenic signaling. Phosphatidylserine also enhances nanoclustering of the KRAS G12D mutant by promoting an alternative type of dimerization that could seed oligomerization [94]. RAS dimerization involves different transient states with the interaction between two α-helices (α:α) being the preferred states [95]. Phosphatidylserine enables KRASG12D mutants to form α:β dimers that could initiate oligomerization and nanoclustering [94]. Interestingly, these dynamic changes in dimer conformations also expose an Achilles heel. The RAS inhibitor BI-2852 binds to a β:β interaction exposed by KRAS G12D resulting in a dimer with an inaccessible effector domain [94]. Thus, RAS dimerization and nanoclustering represent dynamic spatiotemporal mechanisms for regulating RAS signaling that can be exploited as drug targets.

The interactions between RAS nanoclusters and lipids are mutual. Lipids impact RAS nanoclustering and RAS nanoclusters re-order lipids. These relationships have been the topic of recent reviews [96,97], and therefore will not be discussed here.

## Dynamics of RAS–RAF interactions

The discovery that activated RAS directly binds the RAF1 kinase [98–101] was a milestone at several crossroads. It solved the longstanding enigma how RAS proteins exert their biological effects. Simultaneously, it also elucidated a key mechanism how RAF proteins are activated. This direct connection between RAS and RAF signaling spawned decades of intensive research that led to the development of potent RAF inhibitors and to completely new insights into the dynamic wiring and response properties of STNs. Thus, the discussion below is focused on RAS–RAF interactions, but lessons learned can likely be extrapolated to other RAS effector interactions.

The primary direct interaction interface between RAS and RAF are the effector domain and RBD, respectively. RAS to RAF binding induces dimerization of both RAS and RAF [92], thereby mutually enhancing activation and signaling capacity. A close structural examination based on MD showed that the RBD not only induces RAS dimerization but also instigates oligomerization by contacting the scaffold protein Galectin and linking RAS-RBD dimers with each other [102]. Thus, RBD binding triggers a chain reaction of dynamic PPIs that likely culminate in RAS nanoclustering. However, the RAS binding interface in RAF extends beyond the RBD. Directly adjacent, at the C-terminus of the RBD is a cysteine-rich domain (CRD). The CRD is required for full RAF activation, but its role is not entirely clear [103]. The CRD can bind to the lipid tail of RAS, the membrane and also the RAS protein, all with relatively weak affinities [104–106]. It seems that it can dynamically move between these different binding sites, likely to help orchestrating RAS dimerization and RAF activation by relieving RAF autoinhibition. A critical step facilitated by the CRD binding to RAS and/or the membrane is the dephosphorylation of the inhibitory phospho-S259, which is required for RAF1 activation [107]. Interestingly, this dephosphorylation is enabled by another RAS protein, MRAS, which forms a complex with the SHOC2 scaffold and protein phosphatase 1 (PP1) and directs PP1 to phospho-S259 at the PM [108]. The BRAF CRD is frequently mutated in Rasopathies conferring stronger phospholipid binding and better relief of autoinhibition, which translates into enhanced ERK pathway activation [109]. Thus, subtle dynamic changes in the RAS–RAF interaction can cause biologically profound alterations in downstream signaling.

Molecular details how RAF becomes activated when binding to RAS was further elucidated in an elegant MD study that simulated a KRAS nanocluster together with its scaffold protein Galectin-1 and a 14-3-3:RAF1:MEK complex binding to it [110]. Modeling KRAS dimerization confirmed the experimentally observed α:α dimerization [95], but also suggested a new mode of dimerization, GTP-mediated asymmetric (GMA), where one KRAS protein latches on to the γ-phosphate of the GTP-bound by the other KRAS proteins [110]. This concatenated structure protects GTP from hydrolysis and keeps KRAS active. It also allows 8 KRAS molecules to assemble into a helical oligomer. In this scenario, RAF dimerization also enhances RAS oligomerization. As RAF dimerization and activation is stabilized by 14-3-3 proteins [111], their presence in the complex could further enhance RAS oligomerization and activation in a positive feedback loop. Vice versa, this model also could explain how wild-type KRAS can suppress tumorigenesis by oncogenic KRAS mutants in some tissues [112]. Incorporation of a wild-type KRAS protein that is GDP-bound would interrupt the GMA mode of dimerization and stop oligomerization and nanoclustering.

Taken together, these findings suggest close mutual relationships between the activation of RAS and its effector RAF. Other effector interactions are much less well studied, but there is no reason to believe that they are less complex. In the next section, we will discuss how signaling dynamics downstream of RAS are shaped, again mainly using the RAF–MEK–ERK pathway as the best-studied example.

## RAS signaling dynamics shaping downstream signaling

Currently, we know 56 bona fide RAS effectors, which fall into 12 functional classes [5]. They all compete for binding to the same effector domain begging the question of how this is co-ordinated and how it affects downstream signaling. For simplicity, effectors can be divided into high (*K_d _*< 1 μM) and low (*K_d_ *> 1 μM) affinity interactors [5].

**Figure 7.**
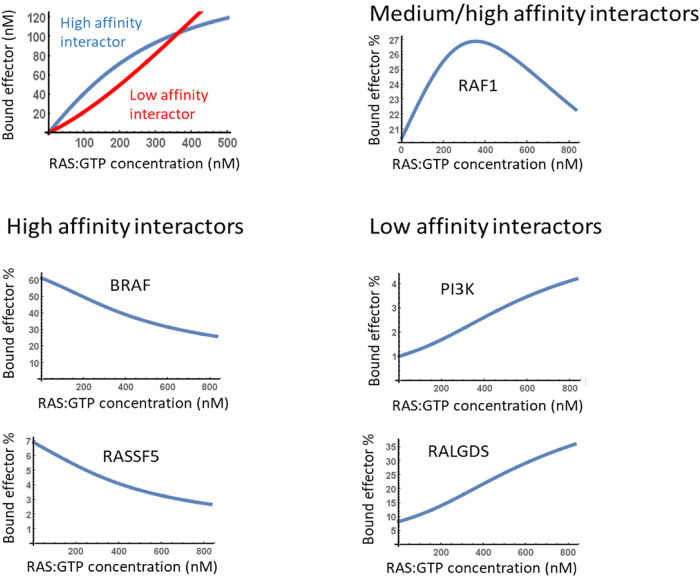
Increasing RAS activity shifts effector binding to low- affinity interactors. Results shown are based on experimental results and a mathematical model of RAS — effector binding reported in [113].

**Figure 8.**
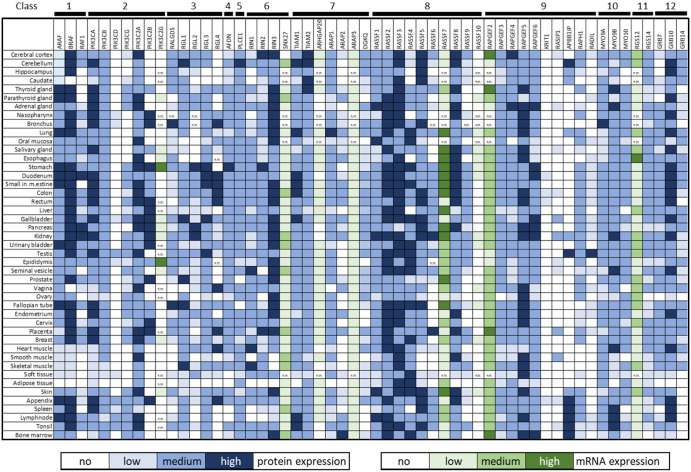
Tissue-specific expression of RAS effectors compiled from the Human Protein Atlas (HPA) [114,115]. Protein expression is given as scored by the HPA. Where protein expression data were not available, mRNA expression data were used. Transcripts per million (TPM) were classified by the following cutoffs: <1 no, 1–10 low, 10–30 medium, and >30 TPM high expression. n.m., not measured.

A simple mass action model shows that high-affinity effectors interact first, but as active RAS concentration increases low-affinity effectors take over [113]. Interestingly, the critical active RAS concentration where the propensity of RAS effector complexes dips towards low-affinity effectors is around the concentration reached when RAS is mutated (Figure 7). This sets up a very dynamic landscape of RAS effectors in RAS-mutated cancers. Assessing the expression of the 56 bona fide RAS effectors in 45 human tissues from the Human Protein Atlas (HPA) [114,115] shows variable effector expression, but also that proteins of each effector class are expressed at medium levels (HPA score) in most tissues (Figure 8). Comparing the expression of the 56 RAS effectors in 29 human tissues to RAS expression showed that taken together the effectors are more abundant than RAS in all tissues except brain and duodenum [116]. This means that in most tissues, effectors compete for binding to activated RAS. Ranking effectors by the expected abundance of RAS effector complexes suggests that six high-affinity effectors (ARAF, BRAF, RAF1, RALGDS, RGL2, RASSF5) constitute up to 80% of RAS effector complexes. Thus, in most healthy tissues RAS signaling would be funneled into three main pathways: RAF, RAL (RALGDS and RGL2 are GEFs for the small GTPase RAL) and RASSF5. Oncogenic RAS mutations increase the concentration of active RAS and shift this balance to include low-affinity interactors, such as Afadin and PI3K. This shift is more pronounced in mutant KRAS than NRAS or HRAS mutants suggesting that oncogenic KRAS signaling may be more malleable by the tissue-specific availability of effectors.

In addition to dynamic signaling changes arising from different RAS isoforms and effector concentrations, clever systems to dynamically manipulate RAS activity with high precision have started revealing the full dynamic capacity of RAS signaling. Using an optogenetic system to bring the catalytic domain of SOS (SOScat) from the cytosol to the membrane results in RAS activation [117]. The advantage of optogenetic systems is their quick response time that allows to shuttle SOScat between cytosol and membrane within minutes. Thus, different kinetic stimulation regimes could be explored using ERK nuclear translocation as readout. The results showed that the RAS–RAF–MEK–ERK pathway functions as a high-bandwidth, lowpass filter. It faithfully transmits stimuli over a large bandwidth, i.e. frequency range, but filters out short-lived stimulation peaks that are likely to represent noise. The ability to apply various kinetic stimulation regimes also proved useful to identify downstream proteins that are differentially activated by short versus sustained RAS signals and which may be involved in decoding transient versus persistent signals [117].

A recent study elegantly combined optogenetics and targeted siRNA screens to identify the network nodes that shape the ERK response dynamics [118]. It used an optogenetically controlled fibroblast growth factor receptor (FGFR) activation system as input and an ERK activity reporter as output. The system could produce rich dynamic behaviors ranging from transient to oscillatory and sustained ERK activation. To identify the nodes that encode the sustained and oscillatory ERK activation regime, 50 nodes were knocked down with siRNA. These nodes included RAS GEFs and GAPs, the core proteins RAS–RAF–MEK–ERK isoforms, adaptor proteins involved in RAS signaling, and transcriptionally induced feedback inhibitors, such as SPRED/SPROUTY and DUSPs. Surprisingly, the knockdown of most nodes only produced subtle effects indicating that the system is robust to perturbations. This robustness is mainly due to two negative feedback loops, one operating from ERK1/2 → RAF1, and the other from ERK1/2 → RSK2 → SOS1. Interestingly, using optogenetic RAS activation (via light-regulated SOScat recruitment to the membrane) did not cause ERK activity oscillations as observed in the optogenetic FGFR system. Moreover, the SOScat system was more sensitive to knockdown perturbations than the FGFR system suggesting that the loss of the ERK1/2 → RSK2 → SOS1 negative feedback also lessens robustness. Eliminating this feedback by RSK2 inhibitors increased the heterogeneity of ERK activation and sensitized the system to the effect of MEK inhibitors.

This is reminiscent of the earlier discovery that the negative feedback from ERK → RAF1 reduces the heterogeneity of ERK activation in a cell population and confers resistance to MEK inhibitors [119]. Theoretical work also shows that the combination of the negative ERK1/2 → RSK2 → SOS1 and positive RAS:GTP → SOS1 feedback cause oscillations in ERK activity [61] consistent with the experimental results described above [117].

Another interesting question is whether different RAS mutations or mutations in downstream RAS effectors impress different kinetics on RAF pathway activities. BRAF mutations blur the temporal resolution of ERK activity triggered by RAS activity pulses controlled by the optogenetic SOScat recruitment system [120]. They cause a delay in ERK activity decline and promote the expression of cyclin D and cell cycle entry under conditions where RAS activity pulses normally would not support cell proliferation. Interestingly, this phenotype is restricted to P-loop BRAF mutations, which in contrast with the common BRAF V600E mutant require dimerization for signaling. Furthermore, P-loop mutants do not exhibit inhibitory autophosphorylation [121]. Thus, the ability of activated RAS to induce RAF dimerization together with the lack of autoinhibition may explain the prolonged activation of the BRAF P-loop mutants.

Another recent study employed ‘RAS-less’ murine embryo fibroblasts (MEFs), where all three RAS genes were knocked out and reconstituted by the expression of single HRAS, KRAS, NRAS, KRAS G12C, KRAS G12D, KRAS G12V, KRAS Q61R genes, or the oncogenic BRAF V600E gene (without RAS gene expression) [122]. ERK activation kinetics were quantified in single cells using the FRET-based EKAR3 probe. Several interesting observations emerged. Wild-type HRAS and all mutant KRAS cell lines exhibited higher baseline ERK activity than wild-type KRAS, with the highest levels observed in KRAS Q61R and KRAS G12C. However, ERK activity still was inducible by EGF in all but the BRAFV600E cells. While fewer mutant KRAS cells responded to EGF, the amplitude of ERK activation was similar in cells expressing wild-type or mutant RAS suggesting that KRAS mutant proteins still are sensitive to growth factor activation and that they do not produce unphysiologically high levels of downstream ERK activity despite different RAS activity levels. Negative feedback activity was weaker in mutant than wild-type KRAS cells arguing against a role for stronger feedback to curb ERK activity in mutant KRAS cells. Analyzing this phenomenon by a mathematical model suggested that yet unknown mechanisms constrain ERK activity in mutant KRAS cells to a bandwidth that is optimal for cell proliferation and transformation. Identifying these mechanisms conceivably could detect new vulnerabilities in RAS signaling that could be used as therapeutic targets.

## Wider network effects of RAS signaling dynamics

Understanding the wider effects of RAS signaling dynamics on downstream networks is pre-requisite for understanding how RAS can reprogram cellular fates in physiological and pathophysiological conditions. Recent advances have analyzed RAS signaling in complex environments and settings of deep and dynamic downstream network analysis. Due to space constraints, we can only discuss illustrative examples.

The above results show that mutant RAS is not an autonomous oncogene but still subjected to modulation by growth factors and the microenvironment. For this, studies in cell systems where RAS drives progressive levels of malignancy are informative. Basal-like breast cancer (BLBC) is an aggressive cancer where tumor cells can adopt multiple states that vary in tumor-initiating capacity and drug resistance [123]. Although BLBCs rarely have RAS mutations, they frequently overexpress the EGFR and show EGFR transcriptional signatures. A BLBC system of non-malignant S1 and T4-2 malignant derivative cells was used in a smart co-culture model to analyze how paracrine signaling can drive heterogeneity in gene expression patterns similar to those found in BLBC. The results suggest that heterogeneity in the tumor microenvironment caused by paracrine growth factor production and stimulation is amplified by the RAS–ERK pathway to result in heterogenous gene expression. Thus, a general oncogenic function of RAS–ERK signaling may be to promote cellular diversity and consequently also tumor cell heterogeneity.

Similarly, measuring ERK activity in patient-derived organoids (PDO) of colorectal cancer (CRC) patients showed that both mutant BRAF, mutant NRAS and mutant KRAS PDOs require EGFR stimulation to proliferate [124]. Presumably, paracrine or autocrine stimulation of the EGFR in PDOs caused oscillatory ERK activity that could be blocked by EGFR inhibitors leaving a small residual ERK activity level driven by the oncogenes. EGFR blockade induced growth arrest, whereas full ERK inhibition achieved by MEK plus EGFR blockade caused cell death. Thus, the decision between growth arrest and cell death was made by small differences in the low range of ERK activity levels. This indicates that oncogene-driven ERK activity suffices to keep tumor cells alive, but higher levels of growth factor-driven ERK activity are needed for proliferation. They also point to an important role for wild-type RAS proteins to support transformation by mutant RAS alleles.

One aspect of this role was revealed in CRC cells. Mutant KRAS can induce apoptosis by interacting with its effector RASSF1A, a tumor suppressor protein that amongst other proapoptotic pathways activates apoptotic MST2–LATS1 signaling [125]. However, mutant Ras also stimulates the production of autocrine growth factors that activate the EGFR, which suppresses mutant KRAS-induced apoptosis. This protection requires the wild-type KRAS allele, which activates AKT and inhibits the MST2 pathway. The pathophysiological relevance of this finding is corroborated by the observation that most advanced human CRCs with KRAS mutations lose MST2 expression, and that the few mutant KRAS CRCs which retain MST2 feature high apoptosis rates [125]. Interestingly, the ability of KRAS to promote MST2-dependent apoptosis hinges on the different activation kinetics of mutant and wild-type KRAS proteins [126]. Sustained KRAS activity caused by mutations or GEF overexpression stimulates proapoptotic MST2 signaling. Transient, EGFR-induced KRAS activation results in AKT-mediated MST2 inhibition and cell survival and proliferation. Thus, the differential activation of PI3K–AKT signaling by chronic versus acute KRAS activation translates into biological cell fate decisions. Downstream of RAS, the competition between MEK and MST2 for binding to RAF1 combined with phosphorylation-dependent affinity changes generate a switch-like mechanism that co-ordinates proliferative signaling through MEK–ERK with proapoptotic signaling through MST2 [127].

## Towards comprehensive RAS signaling networks

Recent proteomics studies mapping the interactomes of wild-type and mutant RAS proteins have extended our view of the wider RAS signaling network [4,128–132]. A summary of the results presents a large network downstream of 43 effector proteins [4]. Mapping the interactors of the direct RAS effectors over three layers of binary interactions identified 2290 proteins, with 441 PPIs in layer 1, 1660 in layer 2, and 16 979 in layer 3. Gene ontology (GO) enrichment analysis suggested that 42% of the biological processes represented are associated with hitherto unknown RAS effector pathway functions. The RAS effector network also showed substantial cross-talk suggesting that RAS signaling occurs via an extensive and well-co-ordinated effector network.

Several studies combined RAS interaction proteomics with CRISPR screens to assess the biological function of the identified interactors [129–131]. Two studies [129,130] used the BioID system, where the bait is fused to a biotin ligase that tags proteins close to the bait with biotin for subsequent affinity purification with streptavidin beads [133]. BioID can detect transient interactions but also may result in false positive identifications.

Using BioID to compare the interactomes of KRAS G12V, NRAS G12V and HRAS G12V produced 477 potential interactors with a substantial overlap between RAS isoforms [129]. A CRISPR screen targeting 474 of these interactors was used to identify proteins that are required for RAS-mediated oncogenic transformation. Here, there was little overlap between the three RAS isoforms. Further filtering for kinases led to the discovery of the Phosphatidylinositol 4-Phosphate 5-Kinase Type-1 Alpha as a KRAS G12V selective interactor, which mediated proliferation and resistance to MEK inhibitors in pancreatic cancer cell lines.

Another study used BioID to compare wild-type HRAS, KRAS, and NRAS with their oncogenic mutants HRAS G12V, KRAS G12D and NRAS Q61K [130]. Of the 1271 proteins identified, only 150 were shared between the three RAS isoforms including know effectors, such as RAF and PI3K. Of these, 42 interacted stronger with mutant RAS proteins, and 130 of these proteins represented new RAS effectors. To define their biological functions a CRISPR screen against these 150 genes was conducted in five cancer cell lines that depend on mutant RAS and four cancer cell lines that do not. Integrating the BioID and CRISPR screening results highlighted five proteins that are known components of RAS PPI networks and whose knockout compromised the viability of multiple RAS-transformed cell lines. One of these was the mTORC2 kinase protein complex, which bound all three mutant RAS isoforms in different cell types. Mutant RAS increased mTORC2 kinase activity which facilitated cell cycle entry and tumorigenesis.

A recent study cast the net wider [131]. Using GTP (G12V) and GDP (S17N) bound mutants of HRAS, KRAS and NRAS in AP-MS experiments, this study constructed a GTP-dependent RAS interaction map that was further expanded by other GTPases (RALA/B, RAP1A/B, RAP2A/B), GEFs (RALGDS, RGL1, RGL2, RASGRF2) and the RAS effector RADIL. The resulting network of 930 proteins showed differences in effector binding, especially to HRAS. For instance, KRAS and NRAS strongly bound to RAF family members, whereas HRAS selectively interacted with RAP GEFs. Furthermore, the RAS and RAP interactomes overlapped extensively, while RALA/B bound to different effectors. The network also revealed extensive functional interactions between RAS, RAP and RAL GTPases mediated by GEFs. Thus, activation of one GTPase could easily spread to others for a concerted response. The interaction proteomics screen was complemented by a dual CRISPR knockout screen targeting 7021 pairs of 119 genes in two mutant KRAS lung cancer cell lines. The target genes were selected based on PPI interaction strengths, a preliminary single CRISPR screen, and published CRISPR screens. Of the 548 and 447 genetic interactions identified in A549 and H23 cells, respectively, only 59 overlapped. This overlap was enriched for paralogs, e.g. RAP1 and RAP2, and genes functioning in the same pathway, such as RAF1 and ERK2. The genetic interaction map confirmed the importance of the RAF–MEK–ERK pathway, but also showed synergistic relationships between RAF1 and the PI3K pathway. Further screening of the strongest genetic interactions in nine lung cancer cell lines uncovered a robust KRAS-dependent interaction between RHOA and RAP1GDS1, a GEF for RHO family GTPases. The double knockout compromised the viability of KRAS-dependent lung cancer cells *in vitro* and in *in vivo* xenografts. This elaborate combined screen identified new potential combination targets for RAS-mutated cancers.

What remains puzzling, however, is the limited overlap between different studies and cell lines. While there usually is a reasonable overlap between strong PPIs and genetic dependence on the nodes involved, there is much less overlap between the synthetic lethal interactions found in different studies. It is currently unclear whether these are technical limitations or a reflection of biological variability. A consistent exception is the RAF–ERK pathway, which seems to be important for RAS transformation in most cells and tissues.

A potentially major source of biological variability is the adjustments RAS-mutated cells have to make to proliferate in the presence of continuously elevated RAS activity. Several lines of experiments suggest that the activity of RAS effector pathways needs to be narrowly controlled for cells to survive and proliferate [10,125,127]. These adjustments were investigated in a pair of KRAS G13D mutated isogenic CRC cell lines, HCT116 and HKE3, that differ threefold in RAS activity [113]. This small difference suffices to render HKE3 non-transformed. It also shifted the balance of RAS interactors from low affinity effectors in HCT116 to high affinity effectors in HKE3. Performing AP-MS on 95 baits in the two cell lines across the known EGFR–RAS network in CRC resulted in two PPI networks with ∼1200 nodes and ∼3000 edges each. Interestingly, the threefold difference in RAS activity caused the rewiring of ∼30% of PPIs. This PPI network rearrangement profoundly affected the signal flow from the EGFR to nuclear transcription factors directing peripheral signals in HCT116 cells towards pro-oncogenic transcription factors, such as FOXO1 and MYC. About 60% of this rewiring could be attributed to differences in protein phosphorylation and protein expression between the two cell lines. Importantly, the most extensively rewired nodes were also frequently altered by mutations and associated with a poor prognosis in CRC patients. Thus, even small changes in RAS activity can trigger widespread functional changes in downstream signaling and biological outcomes.

## Concluding remarks

After four decades of intense research [134] we are still vexed by the intricacies of RAS regulation and functions. However, the fact that what once was the paradigm of an undruggable protein has now become a prominent direct drug target [11] gives hope that we are coming to grips with the intricacies of RAS signaling. This review only focusses on the dynamics of RAS regulation and signaling and, given the extensive literature, even this narrow focus had to be confined to illustrative examples. These concluding paragraphs highlight open questions and possible future directions.

More than 130 RAS mutations are known, while five mutations (G12D, G12V, G12C, G13D and Q61R) account for 70% of all disease-related RAS mutations [135]. However, these numbers still beg the question what is different between these mutations in terms of biological effects. As discussed above, there are salient differences between these RAS mutants in terms of regulation and effector engagement. The main question is whether and how kinetic differences between the upstream regulation and downstream signaling determine biological outcomes. This question has a strong bearing on drug development strategies to target RAS.

Five main themes are emerging. One is the direct targeting of RAS, which has been accomplished for the KRAS G12C allele [136]. However, based on the different functional properties of various mutants, we may need to target different types of RAS mutations with different drugs [11,14].

The second issue arises from the surprising finding that most RAS mutations do not irreversibly lock RAS in the activated state but are still subjected to regulation by GEFs and GAPs. This makes GEFs and GAPs important targets for regulating RAS signaling that would potentially allow to open a therapeutic window based on dynamic differences in GDP–GTP cycling between mutant and wild-type RAS.

The third and related issue is whether and how different RAS mutants use different RAS effectors to produce specific biological effects. We currently know 56 bona fide RAS effectors, which all compete for the same binding site in RAS [4,5]. While high-affinity binders dominate at low RAS activation levels, low-affinity effector complexes become prevalent at high RAS activity levels [113]. This creates a highly dynamic landscape of RAS signaling where quantitative differences in RAS activation are translated to qualitative differences in downstream signaling. One could envision new therapeutic approaches that, rather than trying to inhibit RAS activity, would tune the level of RAS activation or the GDP–GTP cycle to change effector binding from transforming to differentiating or proapoptotic pathways. In this context, it is interesting that the RAF–MEK–ERK, RALGDS–RAL, RASSF, PI3K–AKT and Afadin seem to be the main RAS effector pathways in most tissues based on the predicted abundance of RAS effector complexes [116]. Does this mean that these interactions are carrying out the important RAS functions and that these are the ones we need to target in RAS-driven diseases? Recent CRISPR screens suggest that also other RAS effector pathways are important [129–131,137], albeit there is a little consensus which ones are critical with the exception of the RAF pathway perhaps. The caveat is that we only have studied RAS functions under a limited number of conditions. It is possible that the pleiotropic RAS effectors form a pool of resources through which cells can quickly adapt to different external cues and environmental conditions. This view is supported by observations that mutant RAS signaling is still heavily dependent on and modified by growth factor stimulation [138,139]. Thus, the modulation of RAS signaling by exogenous cues may utilize a much larger array of effectors than the ones we have mapped. Such an adaptive function also is consistent with the tissue-specific expression of many RAS effectors [116], as different tissues experience different environmental cues and therefore have different adaptive requirements.

A fourth aspect is the interconnectivity between different GTPase families that may have the capacity to not only spread and co-ordinate signals but also to buffer perturbations, e.g. drug interference, in the network. RAS signaling is connected to RAL and RHO family GTPases via shared effectors as well as effectors that regulate or function as GEFs and GAPs [131,140–142]. The regulation of RAL (RAL A/B) and RHO (RHO, RAC1/2, CDC42) family members is similar to RAS's. Mediated by GAPs and GEFs they shuttle between a GDP-loaded inactive and GTP loaded active form, which binds to downstream effectors. RHO family members mainly co-ordinate cytoskeletal processes necessary for cell migration [140], while RAL A/B regulate vesicular trafficking and autophagy [141]. These processes generally support RAS-mediated oncogenesis, although RHO proteins have a dual role and also can suppress tumorigenesis [143]. Mapping these GTPase networks will be necessary to understand the full circuitry of RAS regulation and its dynamics.

Finally, we need to solve the role of wild-type RAS alleles in oncogenic transformation by mutant RAS. Wild-type RAS alleles have been reported to both support or suppress oncogenic transformation by mutant RAS [112,125,144,145]. This phenomenon seems to be tissue specific, with wild-type RAS alleles suppressing tumor growth in lung cancer [112], while promoting tumorigenesis in CRC [125]. In KRAS-mutated endometrial cancer the wild-type KRAS allele antagonizes tumorigenesis, whereas wild-type HRAS supports transformation *in vitro* but not *in vivo* [144]. Similarly, in skin cancer the wild-type HRAS allele decreased the number of HRAS mutated papillomas but not the number of squamous cell carcinomas [112]. Evidence suggests that mutant RAS alleles activate the signaling by wild-type RAS alleles due to competition for GAPs [85] or the stimulation of autocrine growth factor production [125,145]. Likely, RAS activation dynamics play a role. For instance, sustained KRAS activation due to mutation or GEF overexpression can engage the proapoptotic MST2 pathway and thereby counteract transformation. Vice versa, transient KRAS activation induces strong AKT signaling which prevents the activation of the MST2 pathway [126].

This type of dynamic interplay may shape the landscape of both physiological and pathophysiological RAS signaling, and a main future challenge will be to decipher how RAS activation kinetics encode different biological information. Accomplishing this task will require a close collaboration between biology and computational modeling which can systematically analyze the dynamic systems behavior [146,147]. Recent advances in computational and mathematical modeling now allow us to investigate RAS pathway dynamics in great detail [138,148], and a new method to assess and control cell states will facilitate the reconstruction and mechanistic analysis of RAS-dependent networks [149].

**Textbox 1. The real Life of Pi — how mathematical and computational models (MCMs) have advanced RAS biology**

The revolutions in physics that changed the world and laid the foundation for much of today's technologies were typically based on MCMs. Copernicus’ and later Kepler's laws of planetary motion pushed the Earth from the center of the Universe to a planet orbiting the Sun. These were nothing but mathematical models that could explain observations enabled by the telescope. However, they profoundly changed how mankind viewed religion, the world, and our role in it. More recently, the discovery of the Higgs boson captured everybody's imagination making headlines for weeks. Again, it was nothing more than an observation consistent with an MCM. On a more practical note, MCMs have been extraordinarily successful in engineering. Any complex piece of engineering nowadays is built, tested and improved in silico first before it is built in real. And while we blindly trust the MCMs that land modern airplanes in zero visibility, we are often skeptical of the value of MCMs in biology and biomedicine. This inconsistency is typically based on a misunderstanding of what an MCM does. 
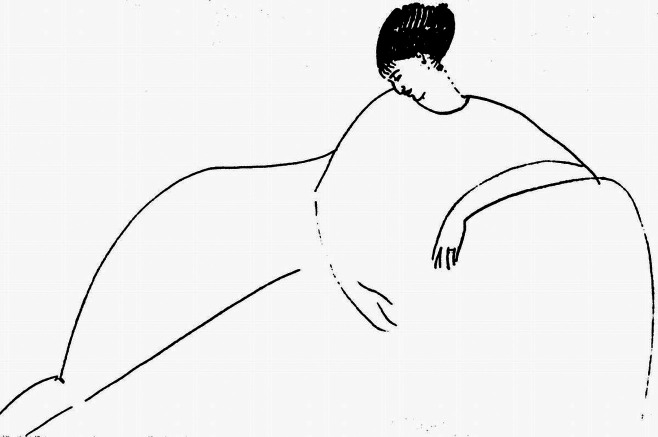

An MCM is not a 1:1 copy of the real world. It is a useful abstraction. ‘Useful’ means that it can explain observations with a certainty that allows predictions of the future behavior of the system and often the understanding of the underlying causal relationships. The essence of a good MCM is that it contains the key elements that characterize the system and separates them from the many other elements that do not matter. This sketch by Modigliani demonstrates the principle: it shows a lady reclining on a sofa in a pensative mood, all captured by a few lines. Thus, MCMs help us to analyze and understand complex systems that transcend human reasoning and intuition. Amongst those are many processes in biology, especially dynamic networks with feedback and feedforward loops, whose behavior is often impossible to understand and predict without MCMs. One recent breakthrough is AlphaFold, which has now become an essential tool in structural biology on par with experimental data [150]. MCMs have also made salient contributions to RAS biology.

More and more detailed examples are discussed in the main text but, in summary, MCMs have contributed to three main areas: (1) *Elucidation of enzymatic reaction mechanisms.* Studying reaction mechanisms at the atomistic level is experimentally difficult and vastly aided by quantum mechanical and MD simulation models [32]. They have resolved distinct steps in the GDP–GTP cycle, how they affect RAS protein conformation [151], and the interaction with effectors [152]. They also facilitate finding drug binding pockets and the design of RAS inhibitors [153]. (2) *RAS activation dynamics.* The amplitude, duration and kinetic shape of RAS signaling is critically dependent on the GDP–GTP exchange cycle and factors controlling it. Early work showed that the transient RAS activation kinetics in response to EGF stimulation not only requires the co-ordinated activation of GEFs and GAPs but also a recruitment mechanism that retains GAPs at the membrane [82]. Later work analyzing the effects of mutations on the GDP–GTP cycle and RAS signaling activity, revealed that RAS mutants that can bind and sequester GAPs induce the activation of wild-type RAS and thereby support transformation [154]. (3) *RAS signaling.* Signaling networks process information, and the activation dynamics of its components is an important way how information is encoded and decoded [6,155,156]. The multiple effectors and complex topologies of RAS signaling networks with intercalated feedback and feedforward loops make their behavior difficult to comprehend through experimentation alone. The surprising failure of potent RAF inhibitors to block RAS signaling is due to dynamic network adaptations and was a key event that rekindled the interest in MCMs [146]. The results are a better understanding of drug resistance and innovative approaches to overcome it [148,157,158]. Notably, some results are unexpected and may not have been discovered without guidance by MCMs. For instance, the synergy between RAF and MEK inhibitors that proved so efficient in melanoma therapy [159] was counterintuitive based on the biological knowledge available at the time. These inhibitors target a linear pathway: MEK is the only known RAF substrate, and both RAF and MEK inhibitors are amongst the most potent kinase inhibitors available. It should be sufficient to inhibit either RAF or MEK, and combining both inhibitors seems making little sense. The explanation lies in lies

the network topology. The RAS–RAF–MEK–ERK module acts as a negative feedback amplifier that effectively buffers perturbations of MEK, which renders MEK inhibitors ineffectual unless the amplifier input is down-regulated by RAF inhibitors [119]. Similarly, MCM showed that combining two structurally different RAF inhibitors can block RAS-mediated transformation [148]. This counterintuitive result is now tested in a clinical trial of KRAS G12D pancreatic cancer (NCT05068752). Thus, it is no surprise that MCMs were recently quoted as a key approach to defeat drug resistance in cancer [160]. Importantly, MCMs also help us to understand complex dynamic processes related to RAS-related GTPase networks that control cell migration, which requires a highly co-ordinated activation/deactivation process of RAC and RHO proteins [161].

## Abbreviations

AP-MS: affinity purification-mass spectrometry
BLBC: basal-like breast cancer
CRC: colorectal cancer
CRD: cysteine-rich domain
DUSP: dual-specificity phosphatase
EGF: epidermal growth factor
ER: endoplasmic reticulum
ERK: extracellular signal-regulated kinase
FGFR: fibroblast growth factor receptor
FRET: Förster resonance energy transfer
GA: Golgi apparatus
GAP: GTPase activating protein
GDP: guanosine-5′-diphosphate
GEF: guanine nucleotide exchange factor
GMA: GTP-mediated asymmetric
GO: gene ontology
GRB2: growth factor receptor-bound protein 2
GTP: guanosine-5′-triphosphate
HPA: Human Protein Atlas
IEG: immediate early gene
LEGI: local excitation/global inhibition
MD: molecular dynamics
MEF: murine embryo fibroblast
MEK: MAPK/ERK Kinase
mTORC: mammalian target of rapamycin complex
NGF: nerve growth factor
NMR: nuclear magnetic resonance
NSCLC: non-small cell lung cancer
PDO: patient-derived organoids
PI3K: phosphoinositide 3-kinase
PIP5K1A: phosphatidylinositol 4-phosphate 5-kinase type-1 alpha
PKC: protein kinase C
PLCγ: phospholipase C-γ
PM: plasma membrane
PP1: protein phosphatase 1
PPI: protein–protein interaction
PTPN12: protein tyrosine phosphatase non-receptor type 12
RAF: rapid fibrosarcoma
RAS: rat sarcoma
RBD: RAS-binding domain
RTK: receptor tyrosine kinase
SHC1: SH2 domain protein C1
SHP2: SH2 domain-containing protein tyrosine phosphatase 2
SOS: son of sevenless
SPRED: sprouty-related EVH1 domain
STN: signal transduction network
